# Activity of daily living upon admission is an independent predictor of in-hospital mortality in older patients with community-acquired pneumonia

**DOI:** 10.1186/s12879-021-06006-w

**Published:** 2021-04-01

**Authors:** Yu Kang, Xiang-Yang Fang, Dong Wang, Xiao-Juan Wang

**Affiliations:** 1 Department of Internal Medicine, Beijing Chao-Yang Hospital, Capital Medical University, 8 Gongren Tiyuchang Nanlu, Chaoyang District, Beijing, 100020 China; 2 Department of Respiratory and Critical Care Medicine, Beijing Chao-Yang Hospital, Capital Medical University, Beijing, China

**Keywords:** Community-acquired pneumonia, In-hospital mortality, Activity of daily living, Barthel index, Older patients

## Abstract

**Background:**

Older patients hospitalized with community-acquired pneumonia (CAP) are at high risk for short-term mortality. Activity of daily living (ADL) is associated with clinical outcomes in older patients. We aimed to investigate the prognostic value of ADL upon admission on the in-hospital mortality in older patients with CAP.

**Methods:**

We conducted a retrospective cohort study involving patients aged ≥65 years admitted to Beijing Chao-Yang hospital due to CAP between June 2012 and June 2020. ADL evaluation upon admission was performed by Barthel Index (BI). Data from all patients were extracted from the electronic medical records.

**Results:**

Four thousand eight hundred eighty patients were included, 131 patients (2.7%) died during their hospitalization. Median BI in the Deceased group was 45 (20–65), Deceased group had lower BI scores than Survivors group (*p* < 0.001). Low BI (< 60) was more frequent in patients who died in the hospital than in patients discharged alive (69.5% vs. 13%, *p* < 0.001). In-hospital mortality was higher among patients with worse ADL upon admission (BI< 60) compared to those BI≥60 (12.6% vs. 0.9%). The worse ADL upon admission (BI< 60) was associated with an increase in the risk of death during CAP hospitalization, worse ADL upon admission (BI< 60) showed an odds ratio (OR) for in-hospital mortality of 7.53 (95%CI: 2.77–20.48; *P* < 0.01). This association remained significant after adjustment for age, comorbid conditions, respiratory failure, pathogens and laboratory findings (OR, 3.74; 95%CI, 2.37–5.91; *P* < 0.01). Receiver operating characteristic (ROC) curve revealed that BI upon admission is a predictor related to in-hospital mortality in elderly patients, the area under the ROC curve of BI in predicting in-hospital mortality was 0.81 (with 95% confidence interval: 0.78–0.85). The predictive value of ADL upon admission was better than age in our study population.

**Conclusion:**

Activity of daily living upon admission is an independent predictor of in-hospital mortality in older patients with community-acquired pneumonia.

**Supplementary Information:**

The online version contains supplementary material available at 10.1186/s12879-021-06006-w.

## Background

Community-acquired pneumonia (CAP) is the commonest cause of infectious death. The incidence and mortality of CAP are linked to increasing age [1, 2]. It is estimated that incidence of CAP patients ≥65 years old was 140 cases per 10,000 persons per year and 105 cases per 10,000 for hospitalized [3]. Mortality of CAP in older patients is more higher than non-elderly patients [3, 4]. Older patients hospitalized with CAP are at high risk for short-term mortality [5]. Thus, there is a clear need to focus on in-hospital mortality in older patients with CAP.

There is growing evidence that the functional status, is more important than age and comorbidity in predicting prognosis in the elderly [6, 7]. Activities of daily living (ADL) assessment is a convenient way to assess a person’s functional level. Even small changes in the ADL functional level are associated with clinically relevant outcomes. ADL functional status decline can lead to adverse clinical outcomes in infections [8–11], acute medical patients [6, 12–14], dementia [15], heart failure [16], hip fractures [17, 18] and geriatric trauma [19]. The ADL functional status may be one of the predictor factor for in-hospital mortality in older patients with CAP. However, there are few data on relationship between ADL and in-hospital mortality in older patients with CAP.

Thus, the purpose of this study is to investigate the prognostic value of ADL upon admission on the in-hospital mortality in older patients with CAP.

## Methods

### Study subjects

We conducted a retrospective study cohort up to hospital discharge. Between June 2012 and June 2020, 4880 patients diagnosed with CAP and age ≥ 65 years were admitted to Beijing Chao-Yang Hospital, Capital Medical University were included in this study. Patients with recent hospitalization or immunosuppressed treatment in the prior 90 days, solid organ or stem cell transplantation in the prior 90 days, cancer with neutropenia or undergoing chemotherapy, tracheotomy were excluded. Beijing Chao-Yang Hospital has the Beijing Institute of respiratory diseases, well-recognized at the national level and the hospital has 1900 beds. All patients underwent ADL evaluation by charge nurse within 2 h after admission. The main outcome was the in-hospital mortality. The patients were divided into Survivors (*n* = 4749) and Deceased (*n* = 131) groups depending on vital status at discharge.

The demographic and clinical information data from all patients were extracted from the electronic medical records. The following variables were collected: age, sex, smoking, co-morbidity, clinical symptoms, clinical conditions and laboratory findings on hospital admission. The study protocol was approved by the Institutional Review Board for Human Studies of Beijing Chao-Yang Hospital, Beijing, China. The informed consent were exempted because this was a retrospective study. Patients’ data confidentiality was fully respected during data collection and the preparation of the manuscript.

### Diagnosis and definitions

The Barthel Index (BI) was used to assess the level of dependency in ADL at the time of hospital admission. The BI measures ten functions that are important for independent living [20]: feeding, dressing, transferring, grooming, bathing, toileting, walking, stair climbing, bowel control, and bladder care. BI score ranging from 0 to 100 points, higher BI score indicates lower dependency. BI < 60 indicates functional depend. The diagnosis of CAP was based on the guideline of Chinese Medical Association (Supplementary Table-1) [21]. CAP was Management in accordance with the IDSA/ATS guidelines [22]. Diagnostic criteria for respiratory failure were as follows: pressure of oxygen (PO_2_) < 60 mmHg under room air according to blood gas analysis on admission or Oxygenation Index (OI) < 300.

### Statistical analysis

Categorical variables were presented as counts and percentages, continuous variables were described by using means and standard deviations and non-normally distributed data were described as median and interquartile ranges. Differences between groups were compared using a Chi-Square test or Fisher’s exact probability test and Student’s *t-*test or Mann-Whitney U test. Logistic regression was performed to assess the relation between ADL and in-hospital mortality. The odds ratios (OR) with 95% confidence intervals (CI) were presented. The receiver operating characteristics (ROC) curves and the areas under the curves (AUCs) were performed to assess the prognostic value. All tests were two-sided, and a value of *P* < 0.05 was considered statistically significant. The statistical analyses were performed by using R software (version 3.3.2) or SPSS 20.0 (SPSS Inc., Chicago, IL, USA).

## Results

### Characteristics of the study population

A total of 4880 patients diagnosed with CAP and aged ≥65 years were included in the study with a median age 72 years (range, 68–80 years) and over half (59.3%) were male. The characteristics of the 4880 patients in the study are shown in Table 1. One hundred thirty-one patients (2.7%) died during their hospitalization. 69.5% patients in Deceased group had a Barthel Index (BI) < 60. Median BI score in the Deceased group was 45 (20–65). Deceased group had lower BI than Survivors group (*p* < 0.001).

**Table 1:** Characteristics of older patients with community-acquired pneumonia

Characteristic	Total patients *n* = 4880	Vital status at discharge		*P* value*
		Survivors *n* = 4749	Deceased *n* = 131	
Age, years	72 (68–80)	71 (67–79)	75 (70–84)	< 0.001
Male, n (%)	2895 (59.3)	2809 (59.1)	86 (65.6)	0.149
Barthel index (BI)	75 (65–85)	75 (65–85)	45 (20–65)	< 0.001
BI< 60, n (%)	720 (14.8)	629 (13.2)	91 (69.5)	< 0.001
BI≥60, n (%)	4160 (85.2)	4120 (86.8)	40 (30.5)	
Comorbid conditions, n (%)				
Smoking	2336 (47.9)	2268 (47.8)	68 (51.9)	0.425
COPD	676 (13.9)	656 (13.8)	20 (15.3)	0.609
Lung cancer	478 (9.8)	455 (9.6)	23 (17.6)	0.004
Diabetes	1214 (24.9)	1172 (24.7)	42 (32.1)	0.037
Chronic heart failure	922 (18.9)	893 (18.8)	29 (22.1)	0.365
Hypertension	2392 (49.0)	2321 (48.9)	71 (54.2)	0.250
Cerebrovascular disease	330 (6.8)	316 (6.7)	14 (10.7)	0.077
Chronic renal failure	86 (1.8)	83 (1.7)	3 (2.3)	0.520
Chronic liver disease	146 (2.9)	145 (3.1)	1 (0.8)	0.188
Clinical symptoms, n (%)				
Fever	1773 (36.3)	1715 (36.1)	58 (44.3)	0.065
Cough and expectoration	1014 (20.8)	980 (20.6)	34 (25.9)	0.155
Chest pain	433 (8.9)	427 (8.9)	6 (4.6)	0.086
Dyspnea	853 (17.5)	816 (17.2)	37 (28.2)	0.002
Duration of symptoms	6.90 ± 8.78	6.86 ± 9.03	6.93 ± 8.21	0.964
Clinical data, n (%)				
Respiratory failure	210 (4.3)	147 (2.4)	63 (48.1)	< 0.001
NIV/IMV	167 (3.4)	115 (2.4)	52 (39.7)	< 0.001
Impaired consciousness	40 (0.8)	28 (0.6)	12 (9.2)	< 0.001
Respiratory rate ≥ 30/min	66 (1.4)	57 (1.2)	9 (6.9)	< 0.001
Blood pressure SBP < 90 mmHg	26 (0.8)	23 (0.5)	3 (2.3)	0.050
DBP ≤ 60 mmHg	594 (12.2)	570 (12.0)	24 (18.3)	0.265
BUN≥7 mmol/L	1531 (31.4)	1405 (29.6)	126 (96.2)	< 0.001
WBC < 4.0 × 10^9^/L or > 10.0 × 10^9^/L	858 (17.6)	807 (17.0)	51 (38.9)	< 0.001
PLT < 10.0 × 10^9^/L	255 (5.2)	210 (4.4)	45 (34.4)	< 0.001
Pathogens, n (%)				< 0.001
Bacterial pneumonia	4144 (84.9)	4050 (85.3)	94 (71.8)	
Viral pneumonia	62 (1.3)	56 (1.2)	6 (4.6)	
Fungal pneumonia	146 (3.0)	129 (2.7)	17 (12.9)	
Mycoplasma pneumonia/Chlamydial pneumonia	528 (10.8)	514 (10.8)	14 (10.7)	

Data are presented as median (interquartile range), mean (standard deviation) or n (%)

*COPD* chronic obstructive pulmonary disease, *IMV* invasive mechanical ventilation, *NIV* non-invasive ventilation, *SBP* Systolic blood pressure, *DBP* Diastolic blood pressure, *BUN* blood urea nitrogen, *WBC* white blood cell, *PLT* platelet

*For comparisons between Survivors group and Deceased group

### The ADL upon admission and in-hospital mortality in older patients with CAP

In-hospital mortality was higher among patients with worse ADL upon admission (BI< 60) compared to those BI≥60 (12.6% vs. 0.9%).

For all clinical presentation presented in Table 1, we initially evaluated each variable that displayed statistical significance with *p* < 0.05 in difference between Deceased and Survivors group using univariate analysis (Table 2). Input variables for the multivariate model were selected from significant variables obtained from the univariate analysis. Dyspnea was not included in multivariate model to eliminate the influence of multicollinearity.

**Table 2:** Logistic regression analysis for in-hospital mortality

Variables	Univariate Analysis		Multivariable Analysis	
	OR (95% CI)	***P*** value	OR (95% CI)	***P*** value
Age	1.70 (1.08–2.66)	0.03	1.58 (0.95–2.63)	0.09
Male	1.01 (0.68–1.49)	0.38		
BI< 60	7.53 (2.77–20.48)	< 0.01	3.74 (2.37–5.91)	< 0.01
Lung cancer	3.05 (2.24–4.14)	0.01	2.38 (1.35–4.21)	0.03
Diabetes	1.54 (1.06–2.23)	0.02	1.26 (0.99–1.59)	0.07
Dyspnea	1.51 (1.19–1.92)	0.04		
Respiratory failure	17.81 (12.04–26.36)	< 0.01	7.54 (3.72–15.27)	< 0.01
Impaired consciousness	1.47 (0.72–3.98)	0.16		
Respiratory rate	1.01 (0.99–1.03)	0.17		
BUN	5.38 (3.16–9.11)	< 0.01	2.61 (1.51–4.52)	0.01
WBC	2.36 (1.42–3.93)	< 0.01	1.68 (1.30–2.17)	0.02
PLT	1.36 (0.87–2.13)	0.11		
Viral or Fungal pneumonia	5.58 (3.16–9.85)	< 0.01	2.25 (1.25–4.05)	0.01

*OR* odds ratio, *Cl* confidence interval, *BI* Barthel Index, *BUN* blood urea nitrogen, *WBC* white blood cell, *PLT* platelet

In logistic regression model (Table 2), the worse ADL upon admission (BI< 60) was associated with an increase in the risk of death during CAP hospitalization, worse ADL upon admission (BI< 60) showed an OR for in-hospital mortality of 7.53 (95%CI: 2.77–20.48; *P* < 0.01). This association remained significant after adjustment for Age, Comorbid conditions, Respiratory failure, Pathogens, White blood cell and Blood urea nitrogen (OR, 3.74; 95%CI, 2.37–5.91; *P* < 0.01).

### Prognostic value of ADL upon admission for in-hospital mortality

**Fig. 1:**
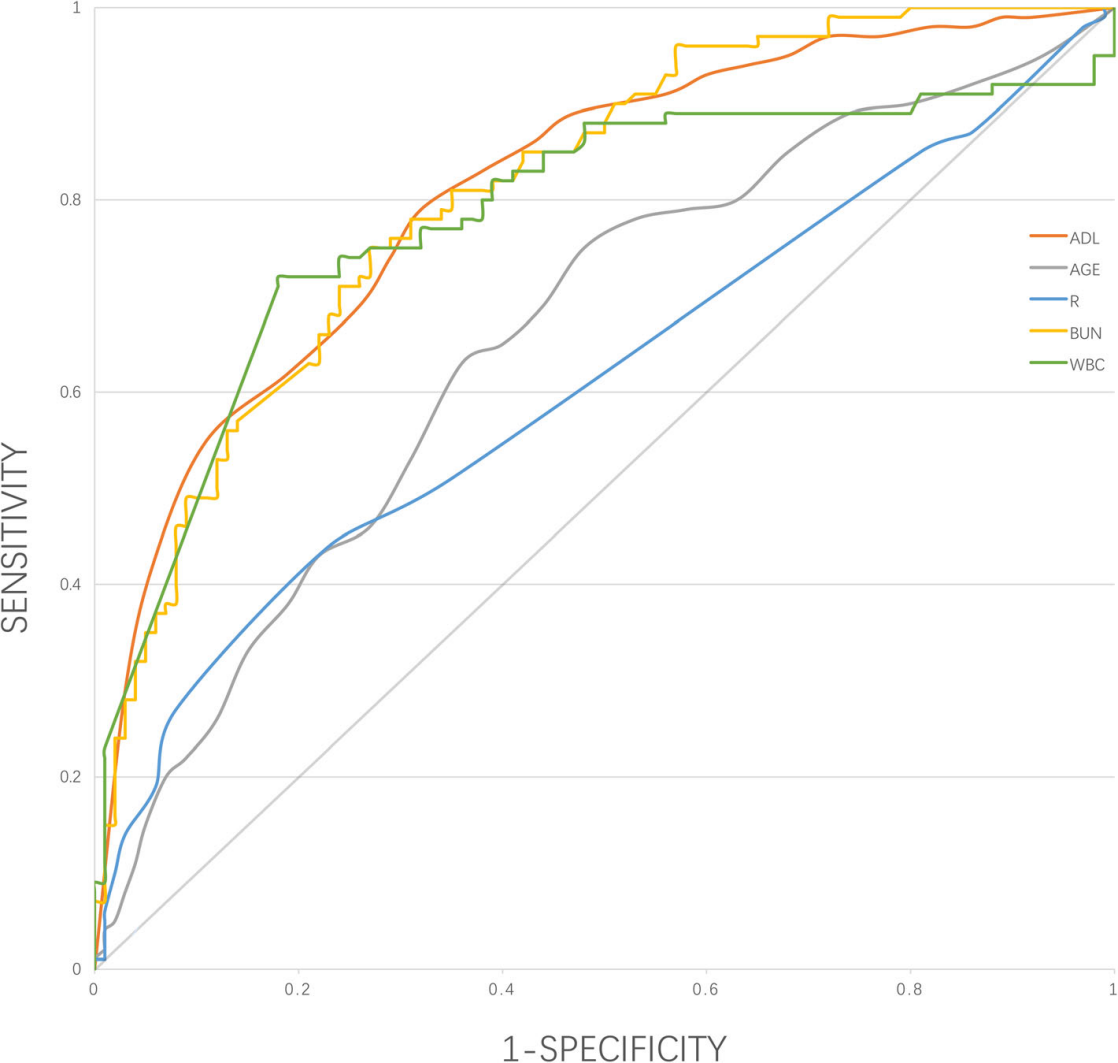
Receiver operating characteristic (ROC) curves for prediction of in-hospital mortality. ADL: activity of daily living. R: respiratory rate. BUN: blood urea nitrogen. WBC: white blood cell

We examined the role of ADL upon admission as a predictor of in-hospital mortality in older patients with CAP. The receiver operating characteristics (ROC) curves and the areas under the curves (AUCs) were performed to assess the prognostic value (Fig. 1). The Barthel Index (BI) was used to assess the level of ADL upon admission. The area under the receiver operating characteristic (ROC) curve of Barthel Index in predicting in-hospital mortality was 0.81 (95% CI, 0.78–0.85). Using Youden Index, the best cut-off point for Barthel Index was 67.5 for in-hospital mortality (sensitivity: 0.79 and specificity: 0.68). The predictive value of ADL upon admission was similar to WBC or BUN, better than age or respiratory rate.

## Discussion

The main finding in this study was the activity of daily living upon admission is an independent predictor of in-hospital mortality in older patients with community-acquired pneumonia. We noted that worse ADL upon admission (BI< 60) was associated with a nearly 7 times increase in the risk of death during hospitalization (OR, 7.53; 95%CI, 2.77–20.48; *P* < 0.01). This association remained significant after adjustment for age, comorbid conditions, respiratory failure, pathogens, WBC and BUN (OR, 3.74; 95%CI, 2.37–5.91; *P* < 0.01). ROC curve revealed that ADL upon admission is an important predictor of in-hospital mortality in older patients. The prognostic value of ADL function at admission was good, as shown by the ROC curve. Hitherto, association between ADL function upon admission and in-hospital mortality in older patients with pneumonia has not been studied, and our study had relatively adequacy sample size. Our study may represent useful information for planning of clinical strategies in older patients hospitalization for CAP.

Older patients hospitalized with CAP are at high risk for short-term mortality [5]. The in-hospital mortality observed in this study was 2.7%. Previous literature reported the in-hospital mortality of 1 to 5% [5, 23–26], with two multicenter studies reporting rates 2.2% [5, 23], our result is consistent with previous literature.

Identifying predictor factors for pneumonia in older patients is crucial in clinical decision making [27–29]. The death of frail elderly with pneumonia is not frequently only due to pneumonia itself [5, 30]. Jason P found that early and aggressive management measures, implemented and valuation of prognosis within 24 h decrease mortality in severe CAP [31]. Different prognostic scales have been documented to assess in CAP, the most commonly be used are the PSI and CURB-65. However, both the PSI and the CURB-65, in contrast to our study data on ADL functional status was lacking. Increasing age is considered to be a factor affecting clinical outcomes in previous study [2, 32]. It is noteworthy that the predictive value of ADL upon admission was better than age in our study. Functional status in elderly patients are frequently individualized [33]. Increasing age does not always mean poor functional status, on the other hand, some older adults experienced a more significant decline in functional status than their peers. The functional status, is more important than age in predicting prognosis in the elderly.

There is growing evidence that poor ADL function is associated with increased mortality. Even little changes in the ADL function could lead to poor clinical outcomes. ADL functional status has been shown to be an independent predictor of mortality in heterogeneous populations [7–19]. In CAP, a worse ADL is directly related to increased short-term and long-term mortality [9]. It was reported that a BI level<80 was associated with 30-day mortality in pneumonia patients [8] and a low BI with increased mortality in institutionalized patients [7]. On the other hand, a high BI level has been reported was related to reduced 30-day and 18 months mortalities in elderly CAP patients [10]. It was found that BI was one of the risk factors for 6 month mortality in COPD patients [11]. While, a worse baseline BI was reported associated with greater mortality in elderly patients admitted to the emergency because of fever [14]. Our study show the same trend as all these previous studies, the worse ADL upon admission (BI< 60) was associated with an increase in the risk of death during CAP hospitalization. Activity of daily living upon admission is an independent predictor of in-hospital mortality in older patients with community-acquired pneumonia.

Assessment of ADL at admission could potentially be used in further management of CAP in older patients. Barthel Index (BI) can effectively performed to evaluate ADL [34]. Barthel Index is a widely used functional assessment of ADL. BI is the official ADL tool of geriatric patients. All patients admitted to ward were evaluated in our hospital. The BI is a simple and rapid assessment tool with high reliability. It takes approximately 5 min to carry out, and it is easy to interpret in clinical practice. The Barthel Index (BI) is reliable, simple, and it can be used as a conventional method for the assessment of the ADL functional status upon admission in patients with CAP.

The study has some limitations. First, this was a single-center retrospective study in an urban area, the potential bias may be due to the single-center design. Secondly, the environment may have some influence on BI. Thirdly, it is unknown if measures such as functional exercise or nutrition supplementation could improve activities of daily living function to improve the prognosis, future research will be required.

## Conclusions

Activity of daily living upon admission is an independent predictor of in-hospital mortality in older patients with community-acquired pneumonia.

## Supplementary Information

**Additional file 1:** **Supplementary Table 1.** Diagnostic criteria for CAP.

## Data Availability

Raw data is available from the corresponding author on reasonable request.

## Abbreviations

CAP: Community-acquired pneumonia
ADL: Activity of daily living
BI: Barthel index
IDSA/ATS: Infectious Diseases Society of America and the American Thoracic Society
OR: The odds ratios
CI: Confidence intervals
ROC: The receiver operating characteristic curve
AUC: The areas under the curves
COPD: Chronic obstructive pulmonary disease
SBP: Systolic blood pressure
DBP: Diastolic blood pressure
BUN: Blood urea nitrogen
WBC: White blood cell
PLT: Blood platelet
IMV: Invasive mechanical ventilation
NIV: Non-invasive ventilation
PSI: Pneumonia severity index
