# Supplementary material for: Activity of daily living upon admission is an independent predictor of in-hospital mortality in older patients with community-acquired pneumonia
Source: BMC Infect Dis. 2021 Apr 1;21:314. doi: 10.1186/s12879-021-06006-w (PMC8017749; doi:10.1186/s12879-021-06006-w)
Supplement: Supplementary file 1 — Additional file 1: Supplementary Table 1. Diagnostic criteria for CAP. [file 12879_2021_6006_MOESM1_ESM.docx]

**Supplementary Table 1.** **Diagnostic criteria for CAP**

| A. Onset in community. |
| --- |
| B. Relevant clinical manifestations of pneumonia |
| (1) New onset of cough or expectoration, or aggravation of existing symptoms of respiratory tract diseases, with or without purulent sputum, chest pain, dyspnea, or hemoptysis |
| (2) Fever |
| (3) Signs of pulmonary consolidation and/or moist rales |
| (4) Peripheral white blood cell count WBC >10 ×10^9^/L or < 4 ×10^9^/L, with or without a left shift |
| C. Chest radiograph showing new patchy infiltrates, lobar or segmental consolidation, ground-glass opacities, or interstitial changes, with or without pleural effusion |
| Clinical diagnosis can be established if a patient satisfies Criterion A, Criterion C and any one condition of Criterion B. Meanwhile, tuberculosis, pulmonary tumour, noninfectious interstitial lung disease, pulmonary edema, atelectasis, pulmonary embolism, pulmonary eosinophilia and pulmonary vasculitis are all excluded. |
